# Sexual Satisfaction Concept Analysis in Iranian Married Women: A Hybrid Model Study

**DOI:** 10.5539/gjhs.v7n6p345

**Published:** 2015-05-20

**Authors:** Zohre Parsa Yekta, Firoozeh Raisi, Abbas Ebadi, Zahra Shahvari

**Affiliations:** 1 School of Nursing and Midwifery, Medical-Surgical Department, Tehran University of Medical Sciences, Tehran, Iran; 2 Roozbeh Psychiateric Hospital Psychiatry & Clinical Psychology Research Center, Tehran University of Medical Sciences, Tehran, Iran; 3 Behavioral Sciences Research Center (BSRC), Nursing Faculty, Baqiyatallah University of Medical Sciences, Tehran, Iran

**Keywords:** sexual satisfaction, marital relationship, Concept Analysis, Hybrid Approach

## Abstract

**Background:**

Sexual satisfaction is considered to be a sexual right and an important component of sexual health. The purpose of this qualitative study was to clarify the meaning and the nature of sexual satisfaction in Iranian married women, and to provide a cultural-based definition of it.

**Method:**

Sexual satisfaction was examined in three phases by the Hybrid Model of concept analysis: (1) the theoretical phase; (2) the fieldwork phase and (3) the analytical phase. Hybrid concept analysis method was chosen because its inclusion of married women’s perspectives enriches the limits of sexual health search literature.

**Result:**

The critical attributes of sexual satisfaction were investigated. They included ‘two-dimensional structure’, ‘an affective response’, ‘a means to achieve marital satisfaction’, ‘unique’, ‘a concept based on expectations’ and ‘a concept on shadow of values’.

**Conclusion:**

The concept analysis of sexual satisfaction showed some of the attributes and antecedents for this concept that, have not been mentioned in the literature.

## 1. Introduction

Sexual satisfaction is considered a sexual right, an important component of sexual health, and a consequence of sexual well-being (WHO, 2010). As a central dimension in the study of relationship quality, it is classified as the barometer for the quality of a relationship (Sprecher & Cate, 2004). The majority of the existing literature on sexual satisfaction lacks a definition of this construct (Ziherl & Masten, 2010). In Higgins’ view (2011) sexual satisfaction has two dimensions: physiological and psychological. Sexual self-comfort, orgasm frequency, and relationship status were significant predictors of high levels of physiological satisfaction among women and unique predictors of high levels of psychological satisfaction were sexual guilt and self-esteem. Haavio-Mannila and Kontula (1997) believe that sexual satisfaction is a two dimensional construct. McClelland (2010) believes that the psychological construct of sexual satisfaction aims to highlight whether a person has reached a level of fulfillment with his/her sexual life, which is an increasingly important consequence in people’s lives. Basson (2001) used the sexual satisfaction concept as a link between arousal and emotional intimacy, and also orgasmic release as a non-essential aspect of sexual satisfaction for women (2000). However, Philippsohn and Hartmann (2009) believe that orgasmic consistency is one of the two dimensions that seem to be central to a woman’s feeling of sexual satisfaction. Women emphasize the emotional aspects of the sexual relationships (Hurlbert, Apt, & Rabehl, 1993). A review of the literature to prompt our understanding of sexual satisfaction is still in its infancy (McClelland, 2010), and needs further development. Sex occurs within a broad social and cultural context (Buss, 2002), while it is essential that the concept of sexual satisfaction be explained properly in the context in which it occurs. Iran is an Islamic country. Islamic culture affects all aspects of Iranian life, and sexual life is no exception. For instance, sexual relationships occur in the context of marriage and monogamy in most people. Extra-marital sexual relationship is anathema for all women in our country. Only 8.5% of women have experienced orgasm in their entire sexual life (Mirtaki, 2004). After all, most women are satisfied in their sexual life (Rahmani, Khoei, & Gholi, 2009; Shahvari, Gholizade & Hosseiny, 2010). It seems the meaning of sexual satisfaction is different in Iranian married women. Therefore, an understanding of the meaning of sexual satisfaction is crucial for married women within the context of Iran. Hybrid concept analysis method was chosen because its inclusion of married women perspectives enriches the limits of sexual heath search literature. Hence, the purpose of this study was to achieve an in-depth understanding of the concept of sexual satisfaction in the social and cultural context of Iranian married women. The main research question was “How do Iranian married women experience sexual satisfaction? And how can this concept be clearly defined?’’

## 2. Methods

The present study used the Hybrid Model of concept development to enrich the limited sexual satisfaction literature including Iranian married women perspectives. Concept analysis is a useful method for refining overused and ambiguous concepts (Walker & Avant, 1995) such as sexual satisfaction. The hybrid model is focused on developing a definition based on specific measurable attributes. It consists of three phases: The theoretical phase, the fieldwork phase and the analytic phase (Schwartz-Barcott & Kim, 2000).

### 2.1 The Theoretical Phase

For a theoretical exploration of sexual satisfaction, the literature was reviewed using the integrative review method. An integrative review is a specific review method that précises past theoretical /empirical literature to supply a more extensive understanding of a particular concept (Whittemor & Knafi, 2005). For finding the related articles, the databases of Medline (PubMed), EBSCO and ProQuest were searched. The key words of sexual satisfaction/dis-satisfaction were used in the abstract and title in articles. Overall, 140 articles published from 1960 to 2014 were retrieved, after review, 35 articles were selected and used for the purpose of this work. In analyzing the concept of sexual satisfaction, the researchers sorted and reviewed the concept-related studies. Literature definitions with respect to differences and similarities were reviewed. Each article was examined according to subject(s), definition of sexual satisfaction, theoretical aspects, dimensions, antecedents and consequences of sexual satisfaction. Qualitative content analysis was used to analyze the results.

### 2.2 The Fieldwork Phase

After an extensive review of the literature, a fieldwork phase was followed in order to empirically elucidate and explore the sexual satisfaction concept in Iranian married women. Exploring in this phase involved empirical validation of the concept using a qualitative research method with a content analysis approach (Beth L Rodgers & Kathleen Astin Knafl, 2000).

#### 2.2.1 Setting and Sample

The researcher conducted 20 interviews with women lived in Tehran. The participants were recruited using the purposive sampling method. Face-to-face, in-depth and semi-structured interviews were conducted in a private setting, considering the participants’ choice. The inclusion criteria were (a) being married (b) living in Tehran (c) living with husband, and (d) having at least 12 months marriage experience. Eligible participants who provided written consent were interviewed. Maximum variation sampling strategy (Patton, 2002) was used to include a broad spectrum of women to assure identifying themes across demographic variations. Four demographic characteristics; age, marriage length, education, and socioeconomic status were anticipated to be relevant to perceptions of sexual satisfaction (Barrientos & Páez, 2006; Haavio-Mannila & Kontula, 1997; Sprecher & McKinney, 1993). Variations in these characteristics were sought during recruitment.

#### 2.2.2 Ethical Consideration

The study proposal was approved by the research council of Tehran University of Medical Sciences. The participants were informed that participation was voluntary and that they could withdraw at any time without being penalized. Conversations were tape-recorded with the participants’ permission. A consent form documenting the participant’s voluntary decision to participate was obtained, after that, the research was fully explained.

#### 2.2.3 Data Collection

The fourth author, as a PhD candidate in reproductive health conducted this phase. Each interview was begun with a general question about the participant’s sexual experiences. Then the interviewer gradually moved to those aspects more directly related to the research inquiry. Narratives were initiated with the questions: “What are some of your sexual experiences that have been satisfying for you?” After initial responses, probes and reflective statements were used to follow the participants’ thoughts. Interviews lasted for 90 minutes on average. The conversations were audiotaped with the permission of the participants. Data was collected from December 2013 to May 2014. Data Saturation was confirmed when the last three interviews did not add new codes, attributes, or domains. The data collected were immediately transcribed verbatim and analyzed using qualitative content analysis. The method of coding according to qualitative content analysis was used to derive categories and themes from the data, which were identified from the first interviews and then tested and revised through analysis of succeeding interviews. To enhance true value and applicability, the researcher establishes the interview guide that was amended by two pilot interviews before it became the formal instrument of data collection. The transcriptions were also checked with participants frequently for accuracy, and the principal investigator reviewed and discussed the entire interview coding to ensure consistency. To increase interviewer reliability and consistency only one researcher collected, translated, and analyzed the data. For each interview, the outcomes were discussed and revisions were agreed upon by members of the research team. The principal investigator also carefully reviewed the entire interview coding to confirm consistency.

#### 2.3 The Final Analytic Phase

In the analytic phase, findings from the fieldwork phase are compared with the theoretical phase data to produce a refined definition of the sexual satisfaction supported by both the literature and the women’s’ perspectives (Schwartz-Barcott & Kim, 2000).The analytic approach used to analyze qualitative data is a version of analytic induction in which new empirical data are continuously compared and contrasted with an initial working definition of the concept. These new data are then used to confirm and/or revise the theoretical idea(s) embedded in the current, working definition. The qualitative data obtained from the participants’ in-depth interviews would help the development of cognition and intuition toward the nature of the concept (Beth L Rodgers & Kathleen A Knafl, 2000). Permanent, comparative analysis was performed all over the entire data-gathering process. In this phase, researchers stepped back from the details of field work and re-examined the data in the light of the preliminary research focus. The final analytic phase was conceptual description through integration of the literature findings and the fieldwork data.

## 3. Results

### 3.1 Theoretical Phase

The first definition of sexual satisfaction was obtained, Hudson writes: (1981) sexual satisfaction is understood to imply the degree of concordance and satisfaction in one’s sexual relationship. Pinney’s (1987) definition is: subjective evaluation of the degree to which a woman is satisfied with her sex life. In DeLamater’s view (1991) sexual satisfaction is: the degree to which a person’s sexual activity meets his or her expectations. Davidson, Darling and Norton (1995) have written: a sense of enjoyment of satisfaction with one’s sexual life is, of course, a highly personal sentiment greatly related to an individual’s past sexual experiences, current experiences, and future aspirations.

Lawrance and Byers (1995) have defined sexual satisfaction in design of interpersonal exchange theory in this way: An affective response arising from one’s subjective evaluation of the positive and negative dimensions associated with one’s sexual relationship. This definition contains both affective and evaluative components and thus distinguishes satisfaction from purely affective constructs such as happiness as well as from purely evaluative constructs such as success. But Sprecher and Cate’s (2004) definition is: the degree to which an individual is satisfied or happy with the sexual aspect of his or her relationship. General sexual satisfaction is basically a (subjective) assessment of one’s sex life independent of sexual activity. Thus, in the case of having no desire for sexual activity, sexually inactive women can well report high levels of general contentment with their sex lives (Philippsohn & Hartmann, 2009). Also, sexual satisfaction is an emotional state that occurs with the fulfillment of individual wishes in the area of sexual life (Ziherl & Masten, 2010). Neto based on Diner’s life satisfaction definition has defined sexual satisfaction as a conscious cognitive judgment of one’s sex life, which depends on one’s judgment standard. In Neto’s (2012) view, satisfaction with sex life can be defined as a global evaluation by the person of his or her sex life. It appears that individuals construct a standard, which they perceive as appropriate for themselves, and compare the circumstances of this sex life to that standard. Hence, this is a subjective judgment rather than a judgment based on some externally imposed objective standard. After analysis the definitions four crucial attributes are evident, that encompass the concept of sexual satisfaction. Other quantitative studies were used to identify the dimensions, determinants, antecedents and consequences of sexual satisfaction.

#### 3.1.1 Attributes of Sexual Satisfaction

Attributes are “those characteristics of a concept which appear over and over again” when the concept is defined (Walker & Avant, 1995). There are four attributes of sexual satisfaction in the literature. Most researchers agree that sexual satisfaction is a ‘two dimensional’ construct: emotional and physical (Basson, 2000, 2001; Haavio-Mannila & Kontula, 1997; Higgins et al., 2011; Hurlbert et al., 1993; McClelland, 2010). This attribute is considered essential for sexual satisfaction. Several researchers agree that sexual satisfaction is a ‘judgment’ (DeLamater, 1991; Hudson et al., 1981; McClelland, 2010; Neto, 2012; Pinney et al., 1987; Ziherl & Masten, 2010). Some researchers have mentioned sexual satisfaction as ‘an affective response’ (Basson, 2001; Davidson et al., 1995; Haavio-Mannila & Kontula, 1997; Lawrance & Byers, 1995; Sprecher & Cate, 2004). Other researchers have mentioned ‘sexual satisfaction as an evaluation / assessment’ (Lawrance & Byers, 1995; Neto, 2012; Philippsohn & Hartmann, 2009).

#### 3.1.2 Consequences of Sexual Satisfaction

Sexual satisfaction predicts marriage stability (Heiman et al., 2011), quality of life (Walters & Williamson, 1998), relationship stability and satisfaction with relationship (Sprecher & Cate, 2004), love (Hendrick & Hendrick, 2004). Higher sexual satisfaction is also associated with fewer mental health issues, such as depression or anxiety (Frohlich & Meston, 2002), and with better self-rated physical health (Laumann et al., 2006).

#### 3.1.3 Working Definition

The definition of sexual satisfaction based on these critical attributes is: *A feeling of gratification arising from high concordance of physical and emotional dimensions of sexual life with the already preformed criteria of the individual*.

### 3.2 The Fieldwork Phase

The attributes of sexual satisfaction concept in Iranian married women were extracted as the following: ‘subjective and private’, ‘unique’, ‘a means to achieve marital satisfaction’, ‘two-dimensional structure’, ‘ an affective response’, ‘a concept on shadow of values’ and ‘a concept based on expectations’. Factors (antecedents) that influenced sexual satisfaction were ‘context constructive variable’, ‘individual’s variable of woman’, ‘husband’s variables’, ‘relationship variables’ and ‘safe and appropriate condition’.

#### 3.2.1 Attributes of Sexual Satisfaction

- Subjective and Private
  It is remarkable that in the present study, participants deem sex as a private part of their lives.
  *“Sex is a private part of every one’s life and no one likes to speak about this relationship and about her satisfaction. This subject is so subjective that one cannot even admit satisfaction or dissatisfaction to herself, and this could be affected by daily events. When a woman is in conflict with her husband, even the woman herself could not recognize whether she is satisfied sexually or not”* (46 years old, 15 years married and a worker)
- Unique
  Some women consider sexual satisfaction as a concept that may be defined differently by various people.
  *“Sexual satisfaction could be described considering some conditions. I think sexual satisfaction has a different meaning in youth. I am not young anymore and my body’s hormones work differently. When such serenity and support could be met by my husband, I have no conflict and this never bothers me. This is the condition which defines satisfaction for me…”* (43 years old, 4 years married and a collegian)
- A means to achieve marital satisfaction
  *“In a married life, it is not just a sexual relationship, it is living with another person who is with you every night and every day, you have such a relationship for just one hour. When you like your husband, you like his personality, you like your living condition, and your husband in all things is similar to you; that relationship is affected and can be joyful … I am totally satisfied with my marital life and sex is a part of my marital life, it means I am satisfied with it too”* (26 years old, 4 years married, and a housekeeper)
  “I think 10% of marital relationship is sex, but this 10% could complete the remaining 90%. In a common life, living issues capture 90% of our brain and 10% of it is filled with sex. If you have good sex, just this 10% could solve many problems and other difficulties can be managed ….”(58 years old, 30 years married, and retired)
- Two-dimensional structure
  Participants in this research defined sexual satisfaction as a two-dimensional structure with physical and emotional dimensions. Meeting sexual requirements and its pleasurableness could constitute physical structure of sexual satisfaction. Its emotional dimension was defined as mind/ mental peace, intimacy with husband, and satisfaction with husband’s emotional expression as well as satisfaction with his behaviors in married life.
  *“Sexual satisfaction means one feels delight in everything and thinks nothing is better than this relationship. Then, she will bear all troubles. There will be a feeling of serenity and intimacy with her husband”* (44 years old, 25 years married, and a housekeeper)
  *“Sexual satisfaction means just the feeling that my mind is free from problems along with physical pleasure. If sex is based on principles, its pleasure is beyond any other pleasure, thus it can be defined as a pleasure from sexual tendencies besides mental serenity and such sex is so rare”* (27 years old, 3 years married, and a collegian)
- Affective response
  According to the participants, sexual satisfaction is an emotional response that means satisfaction and feeling of happiness, rather than a judgment, subjective evaluation, assessment and/or compatibility degree. That feeling of contentment and happiness is an emotional response, and then it is a product.
  *“Sexual satisfaction means happiness from sex with your husband. It means that you have a good feeling about your relationship with him. It is the pleasure of being together. A sense of being elated”* (19 years old, 1 years married, and a housekeeper)
- A concept on shadow of values
  Some special values may rule on even a family atmosphere and in the sexual relationship of a couple, in such a way that no convincing reason for some activities can be found. Sexual satisfaction in some Iranian women has been affected by their ideal values, since God’s gratification is the sole motive for them to form a sexual relationship with their husbands.
  *“If a woman could satisfy her husband sexually without asking any remuneration, she has transacted with God. If I, as a woman, know that everything I do at home is not useless and God observes them, I will do them as a tradition without any physical expectation and I am sure that I am praying and I deal with my God and He will respond elsewhere in my life; if I am not rewarded in this life, I will rewarded in the other world …”* (46 years old, 24 years married, and housekeeper)
  Some of the participants deem their ability to meet their husbands’ needs as their abilities and it was so extremely satisfying for them.
  *“It is so enjoyable for me to know that I have a role in my husband’s joy and he pays attention to me and my role. Even if I do not orgasm, my husband’s sexual satisfaction is sufficient for me. This ability to gratify him seems to be enough. Effect of a wife on her husband’s sexual satisfaction is her power”* (38 years old, 10 years married, and a university professor)
- A concept based on expectations
  Expectations in women’s life influence their satisfaction all throughout their lives. Contented people are satisfied with the minimum and exigent ones expect the best from everything.
  “I was contented and my expectations were low. May be my lack of knowledge made me so. From the very first days of my married life, I expected little from our sex … hence, I was not dissatisfied, but now, when I look back through my past, I didn’t have a good sexual life …”

#### 3.2.2 Consequences of Sexual Satisfaction in Fieldwork

Fieldwork phase referred to psychological security, lack of emotional insecurity, adapting with the hardships of life, increased love and affection, loyalty, increased intimacy, remission, peace, tendency to continue sex and marriage.

#### 3.2.3 Features Derived From the Review of the Literature and Fieldwork

‘Two-dimensional structure’, ‘judgment’, ‘affective response’ and ‘evaluation/assessment’, ‘subjective and private’, ‘unique’, ‘means to achieve marital satisfaction’, ‘product’, ‘a concept based on expectations’, and ‘a concept on shadow of values’ are the attributes/ themes derived from the concept of sexual satisfaction in Iranian married women.

#### 3.2.4 Practical Definition

*Sexual satisfaction in Iranian married women includes gratification of engaging in sex and continuing sex with husband mostly for the fulfillment of wife and/or husband’s sexual needs and/or achieving supreme values in/of life, in a way that marital satisfaction is formed and continued. Women’s contentment with their husband’s and their own sexual role and the interactions between them are a central part of sexual satisfaction*.

## 4. Discussion

Because of the importance of sexual satisfaction in married women, the concept analysis approach was used to clarify the concept of sexual satisfaction. Data analysis revealed six attributes for sexual satisfaction, ‘subjective and private’, ‘unique’, ‘a means to achieve marital satisfaction’, ‘product’, ‘a concept based on expectations’ and ‘a concept on shadow of values’.

Some of the attributes achieved in the fieldwork phase are compatible with those of the theoretical phase. According to the participants, sexual satisfaction is an emotional response, feeling of contentment and happiness, and it is a product. Also, some researchers consider it an emotional response and a product.

‘Judgment’, including subjective evaluation, assessment and/or compatibility degree, was an attribute derived from the review of the literature. In literature, the main focus was on the already performed criteria of the individual. However, in the fieldwork phase, it was revealed that there is no specific criterion from future sex in Iranian married women’s minds and whatever occurred in their sexual lives may be found as priority through their married lives and by passing their common lives. One exception is the romantic part of their sexual lives, of which almost all Iranian women before marriage expect to have a romantic life in future. In the review of the literature, having correct judgment, for example, in dimension of compatibility degree are the features of sexual satisfaction. Therefore, this feature should be considered in the sexual training programs for women. Some women consider sexual satisfaction as a ‘unique’ concept. This attribute could be considered a product of judgment that is extracted from other studies. Another attribute was ‘subjective and private’, which was put forward as a theme by most of the participants. Reaching Iranian women’s thoughts and extracting the concept of sexual satisfaction was a hard task (Nasimi & Mahdavi, 2008). Most women considered sex as a part of general married life and ‘a means to achieve marital satisfaction’ and they expected their husbands to look at the issue from the same point of view. They think that married life satisfaction is so important in developing sexual satisfaction and they expected their husbands to play their role perfectly and to be responsible in their lives. Also in the Taiwanese women’s experiences of sexual satisfaction during pregnancy, one of the essential themes was: strengthening the marital bond (Lee, 2002). In the context of Iran, a woman has sex in her marital life. Sexual satisfaction may also be gained in this relationship, and for them continuation of marriage is the main purpose of sex.Antecedents influencing sexual satisfaction include expectations and values.

*Expectations:* Sexual satisfaction is considered ‘a concept based on expectations’ by some participants. Level of expectation, as a women personality characteristic had a significant role in their satisfaction. In the social life, higher expectation was related to more probability of dissatisfaction (Birenbaum & Lesieur, 1982).

*Values:* Among Iranian women, ‘values’ play the main role in sexual satisfaction and sexual satisfaction is a concept on shadow of values. Values could change the expectations in a sexual relationship. They utilize ‘values’ as a moderator for controlling the effects of unpleasant factors on their sexual satisfaction. It means women could tolerate such factors and married life will be continued because of the values governing the sexual relationship. Some values have a role either in sexual satisfaction antecedents or consequences. Feeling sexual empowerment, emotional interest or fondness are examples of these values. For example, feeling sexual empowerment in the current sex is essential for successful start of the next relationship. Feeling of wife’s sexual empowerment was termed ‘the power of women’ by Khoei, Whelan & Cohen (2008). According to Nasimi and Mahdavi, some Iranian women are looking for their husband’s love. A number of them were able to satisfy their husband sexually, and their sexual satisfaction appears to follow the husband’s consent (2008). Actually obtaining husband’s love and his sexual satisfaction creates a sense of empowerment in women and are considered as values for women. A small number of women stated that they responded positively to their husbands’ request just for God’s sake. These women supposed themselves to adhere to religion, and were willing to do what great figures of Islam say about men and women’s responsibility in marriage. Also, they had complete faith in compliance (*Tamkin*) which is emphasized in Islam.

Study of sexual satisfaction leads us to the following findings: Sexual satisfaction is a subjective, private and unique concept with two physical and emotional dimensions. It is a concept whereby women’s expectations from marriage/sexual life and values have effects on its formation. Moreover, the continuation of sex and marriage with husband and emotional serenity and compatibility with life’s hardship are the most significant consequences in Iranian society.

## 5. Conclusion

The attributes of the concept of sexual satisfaction were identified in the research findings. This study provides insight into married women’s’ sexual satisfaction. Moreover, identification dimensions of sexual satisfaction helps the sex therapists in properly diagnosing sexual dis-satisfaction in women with an insight based on scientific findings. Our findings can be a baseline for future research to increase sexual satisfaction in the married women in Iran by development of effective interventions. This study aimed to achieve a deeper understanding of the sexual satisfaction concept in Iranian married women and in any context where permanent marital/sexual relationship is the dominant culture, so that one can develop a questionnaire for measurement of sexual satisfaction in married women that is culturally appropriate.

### 5.1 Study Limitations

This research has limitations of qualitative study such as small sample size, which limits its generalizability to all Iranian women.

## References

[ref1] Barrientos J. E, Páez D (2006). Psychosocial variables of sexual satisfaction in Chile. Journal of sex & marital therapy.

[ref2] Basson R (2000). The female sexual response: A different model. Journal of Sex & Marital Therapy.

[ref3] Basson R (2001). Using a different model for female sexual response to address women’s problematic low sexual desire. Journal of Sex & Marital Therapy.

[ref4] Birenbaum A, Lesieur H (1982). Social values and expectations. The sociology of deviance.

[ref5] Buss D. M (2002). Human mate guarding. Neuroendocrinology Letters.

[ref6] Davidson J. K, Darling C. A, Norton L (1995). Religiosity and the sexuality of women: Sexual behavior and sexual satisfaction revisited. Journal of Sex Research.

[ref7] De Lamater J (1991). Emotions and sexuality. Sexuality in Close Relationships.

[ref8] Frohlich P, Meston C (2002). Sexual functioning and self-reported depressive symptoms among college women. Journal of Sex Research.

[ref9] Haavio-Mannila E, Kontula O (1997). Correlates of increased sexual satisfaction. Archives of sexual behavior.

[ref10] Heiman J. R, Long J. S, Smith S. N, Fisher W. A, Sand M. S, Rosen R. C (2011). Sexual Satisfaction and Relationship Happiness in Midlife and Older Couples in Five Countries. Archives of Sexual Behavior.

[ref11] Hendrick C, Hendrick S. S, J. H Harvey, A Wenzel, S Sprecher (2004). Sex and romantic love: Connects and disconnects. The handbook of sexuality in close relationships.

[ref12] Higgins J. A, Mullinax M, Trussell J, Davidson J. K, Moore N. B (2011). Sexual satisfaction and sexual health among university students in the United States. Journal Information.

[ref13] Hudson W. W, Harrison D. F, Crosscup P. C (1981). A short-form scale to measure sexual discord in dyadic relationships. Journal of Sex Research.

[ref14] Hurlbert D. F, Apt C, Rabehl S. M (1993). Key variables to understanding female sexual satisfaction: An examination of women in nondistressed marriages. Journal of sex & marital therapy.

[ref15] Khoei E. M, Whelan A, Cohen J (2008). Sharing beliefs: What sexuality means to Muslim Iranian women living in Australia. Culture, health & sexuality.

[ref16] Laumann E. O, Paik A, Glasser D. B, Kang J.-H, Wang T, Bernard Levinson M, Alfredo Nicolosi M (2006). A cross-national study of subjective sexual well-being among older women and men: findings from the Global Study of Sexual Attitudes and Behaviors. Archives of sexual behavior.

[ref17] Lawrance K, Byers E. S (1995). Sexual satisfaction in long-term heterosexual relationships: The interpersonal exchange model of sexual satisfaction. Personal Relationships.

[ref18] Lee J. T (2002). The meaning of sexual satisfaction in pregnant Taiwanese women. Journal of Midwifery & Women’s Health.

[ref19] McClelland S. I (2010). Intimate justice: A critical analysis of sexual satisfaction. Social and Personality Psychology Compass.

[ref20] Mirtaki M (2004). Exploring the relationship between orgasm experience and marital satisfactionin the women referring to healthcare centers [MS thesis].

[ref21] Nasimi M, Mahdavi S. M. S (2008). The sociological study of women’s marital relationship satisfaction [Article in Persian]. Journal of social sciences.

[ref22] Neto F (2012). The Satisfaction With Sex Life Scale. Measurement and Evaluation in Counseling and Development.

[ref23] Philippsohn S, Hartmann U (2009). Determinants of sexual satisfaction in a sample of German women. The journal of sexual medicine.

[ref24] Pinney E. M, Gerrard M, Denney N. W (1987). The Pinney sexual satisfaction inventory. Journal of Sex Research.

[ref25] Rahmani A, Khoei E. M, Gholi L. A (2009). Sexual satisfaction and its relation to marital happiness in Iranians. Iranian Journal of Public Health.

[ref26] Rodgers B. L, Knafl K. A (2000). Concept development in nursing: Foundations, techniques, and applications.

[ref27] Schwartz-Barcott D, Kim H. S (2000). An expansion and elaboration of the hybrid model of concept development. Concept development in nursing: Foundations, techniques, and applications.

[ref28] Shahvari Z, Gholizade L, Hosseiny S. M (2010). Determination of some related factors on women sexual satisfaction Gachsaran (south-west of IRAN). Journal of Gorgan university of medical sciences.

[ref29] Sprecher S, Cate R. M (2004). Sexual satisfaction and sexual expression as predictors of relationship satisfaction and stability. The handbook of sexuality in close relationships.

[ref30] Sprecher S, McKinney K (1993). Sexuality.

[ref31] Walker L, Avant K (1995). Strategies for Theory Construction in Nursing.

[ref32] Walters A. S, Williamson G. M (1998). Sexual satisfaction predicts quality of life: A study of adult amputees. Sexuality and Disability.

[ref33] Whittemore R, Knafl K (2005). The integrative review: updated methodology. Journal of Advanced Nursing.

[ref34] WHO (2010). Measuring sexual health: Conceptual and practical considerations and related indicators.

[ref35] Ziherl S, Masten R (2010). Differences in predictors of sexual satisfaction and in sexual satisfaction between female and male university students in slovenia. Psychiatria Danubina.

